# Association of IGF‐1 gene rs2195239 polymorphism with the risk and clinical features of gastric cancer in a Chinese Han population

**DOI:** 10.1002/jcla.23284

**Published:** 2020-03-09

**Authors:** Pengli Wang, Xiaoyan Zhou

**Affiliations:** ^1^ Department of Oncology Affiliated Hospital of Shaanxi University of Traditional Chinese Medicine Xianyang China

**Keywords:** case‐control study, gastric cancer, IGF‐1

## Abstract

**Background:**

Recently, a Japanese study investigated the relationship between insulin‐like growth factor‐1 (IGF‐1) gene rs2195239 polymorphism and gastric cancer (GC) risk and found rs2195239 polymorphism did not relate with the risk of GC. However, no Chinese studies have addressed this relationship until now. Thus, the aims of this study were to demonstrate whether IGF‐1 gene rs2195239 polymorphism was linked with the risk and clinical features of GC in a Chinese Han population.

**Methods:**

In order to verify the link between IGF‐1 gene rs2195239 polymorphism and GC risk, we recruited 361 GC cases and 418 controls in this case‐control study. The genotyping was done by use of a custom‐by‐design 48‐Plex SNP scan TM Kit.

**Results:**

This study found that IGF‐1 gene rs2195239 polymorphism decreased the risk of GC. Stratified analyses suggested that the significant association was shown in the females, non‐smokers, non‐drinkers, and age <60 years groups for GC. In addition, IGF‐1 gene rs2195239 polymorphism correlated with the tumor size, tumor clinical stage, and pathological types for GC patients.

**Conclusion:**

To sum up, this study shows that IGF‐1 gene rs2195239 polymorphism is associated with the risk and clinical features of GC patients in this Chinese population.

## 1 INTRODUCTION

Gastric cancer (GC) is one of the main causes of cancer‐related death worldwide.^1^ More than 950 000 new GC patients are reported every year.^1^ GC is separated into true gastric adenocarcinomas and gastro‐esophageal junction adenocarcinomas anatomically.^2^ GC ranks the 3rd most comment cancer death in China.^3^ Up to date; the pathogenesis of GC remains unclear. Previous studies indicated that *H pylori* infection, smoking, low consumption of fruits and vegetables, alcohol, and high salt intake contribute to the risk of GC^4^, ^5^ However, not all GC patients had these exposed factors, indicating that other aspects including genetic factors may also affect the carcinogenesis of GC.^1^, ^6^

The insulin‐like growth factor (IGF) signaling pathway is a regulatory system including IGF‐1, IGF‐2, the two IGF receptors (IGF‐1R and IGF‐2R), and the six binding proteins (IGFBP1‐6). This signaling pathway was reported to stimulate cell proliferation and inhibits apoptosis.^7^ Animal experiments suggest that IGF‐I may promote tumorigenesis.^8^ IGF‐1 is located on 12q22–24.1. Studies have demonstrated that genetic variations in IGF‐1 gene influence the IGF‐1 levels.^9^ Shitara et al^10^ indicated that variants in IGF‐1 gene were associated with the GC prognosis. Several studies investigated the relationship between rs2195239 polymorphism in IGF‐1 gene and different cancer risks^11^, ^12^, ^13^ including GC.^14^ However, Ennishi et al^14^ did not obtain significant results in this Japanese population. In addition, no Chinese study investigated the link between rs2195239 polymorphism and risk of GC in Chinese individuals previously. In order to uncover this potential association, we designed this hospital‐based case‐control study in a Chinese Han population.

## 2 PATIENTS AND METHODS

### 2.1 Subjects

In this case‐control study, 361 newly diagnosed and histologically confirmed GC patients and 418 sex‐ and age‐matched controls were recruited from the Affiliated Hospital of Shaanxi University of Traditional Chinese Medicine (Xianyang, China). The individuals with gastritis, other cancers, or gastric ulcers were excluded from this study. The control groups were individuals receiving health examinations in the hospital at the same period.

The clinical information including age, sex, smoking status, and alcohol was collected from each participant through a structured questionnaire. Smokers and drinkers were the individuals who smoked one cigarette every day for more than 1 year and those who drank at least twice every week for over half a year, respectively. Clinical information including the *H pylori* infection, TNM stage, tumor size, localization, differentiation, metastasis, and histology was obtained from the medical record. The study was approved by the Ethics Committee of the Affiliated Hospital of Shaanxi University of Traditional Chinese Medicine, and it met the standards of Declaration of Helsinki. Written informed consent was obtained from each subject.

### 2.2 Blood sampling and genotyping

According to manufacturer's instructions, genomic DNA of peripheral leukocytes was extracted using the TIANamp Blood DNA Kit (Qiagen). Rs2195239 polymorphism was genotyped by use of a custom‐by‐design 48‐Plex SNP scan TM Kit (Genesky Biotechnologies Inc.). About 10% of selected samples were validated with direct sequencing to verify the genotyping accuracy.^15^, ^16^ The concordance of genotypes in the repeated samples was 100%.

### 2.3 Statistical analysis

Using the chi‐square test, the differences in frequency distributions of dichotomous variable between cases and controls were evaluated. The Hardy‐Weinberg equilibrium (HWE) for IGF‐1 gene rs2195239 polymorphism was evaluated among the control individuals.^17^, ^18^, ^19^ Logistic regression models were used to address the relationship between rs2195239 polymorphism and GC risk. Odds ratios (ORs) and their 95% confidence intervals (95% CI) were figured out without or with adjustment for age and sex. Subgroup analyses were conducted by sex, age, smoking, and alcohol. The associations between IGF‐1 rs2195239 polymorphism and clinical characteristics of GC were also explored. All analyses with *P* < .05 value were significant. SPSS 22.0 software (SPSS Inc.) was used to deal with all statistical analyses.

## 3 RESULTS

### 3.1 Characteristics of the study population

The demographic data of all participants are shown in Table 1. A total of 361 GC patients with age of 56.25 ± 5.99 years and 418 healthy controls with age of 56.42 ± 6.73 years were enrolled in this study. For cases and controls, the distribution of age and sex had no significant differences. The percentages of smoking, *H pylori* infection, and drinking were higher in the case groups compared with the control groups. Clinical information of GC patients including the tumor size, R classification, Lauren classification, metastasis, histological grade, TNM, tumor location, and histology is presented in Table 1.

**Table 1:** Patient demographics and risk factors in gastric cancer

Characteristics	Case (N = 361)	Control (N = 418)	*P*
Age	58.50 ± 10.11	58.71 ± 9.73	.760
Sex			
Male	192 (53.2%)	225 (53.8%)	.858
Female	169 (46.8%)	193 (46.2%)	
Smoking			
Yes	250 (69.3%)	219 (52.4%)	<.001
No	111 (30.7%)	199 (47.6%)	
Alcohol			
Yes	256 (70.9%)	210 (50.2%)	<.001
No	105 (29.1%)	208 (49.8%)	
*H pylori*			
Seronegative	115 (31.9%)	217 (51.9%)	<.001
Seropositive	246 (68.1%)	201 (48.1%)	
Tumor size			
>4 cm	183 (50.7%)		
≤4 cm	178 (49.3%)		
R classification			
R0	101 (28.0%)		
R1	204 (56.5%)		
R2	56 (15.5%)		
Lauren classification			
Intestinal	217 (60.1%)		
Diffuse	139 (38.5%)		
Mixed	5 (1.4%)		
Metastasis			
M0	337 (93.4%)		
M1	24 (6.6%)		
Histological grade			
Well differentiated	57 (15.8%)		
Moderately differentiated	189 (52.4%)		
Poorly differentiated	115 (31.9%)		
TNM			
Ⅰ	88 (24.3%)		
Ⅱ	120 (33.2%)		
Ⅲ	88 (24.4%)		
Ⅳ	65 (18.1%)		
Location			
Cardia	106 (29.4%)		
Non‐cardia	255 (70.6%)		
Histology			
Adenocarcinoma	344 (95.3%)		
Not Adenocarcinoma	17 (4.7%)		

Note

R0: No cancer infiltration at the margin; R1: Microscopic cancer infiltration; R2: Macroscopic cancer infiltration.

### 3.2 Association between IGF‐1 gene rs2195239 polymorphism and GC risk

Using logistic tests, the link between IGF‐1 gene rs2195239 polymorphism and GC susceptibility was analyzed in five genetic models (Table 2). The rs2195239 polymorphism met with HWE. In this study, data showed that the GG genotype or G allele was related with decreased risk for GC (GG vs CC: OR, 0.59; 95% CI, 0.36‐0.97; *P* = .036; G vs C: OR, 0.77; 95% CI, 0.62‐0.95; *P* = .015). After adjusting for sex and age, these associations were still significant.

**Table 2:** Genotype frequencies of IGF‐1 gene polymorphisms in cases and controls

Models	Genotype	Case (n, %)	Control (n, %)	OR (95% CI)	*P*‐value	^a^OR (95% CI)	^a^ *P*‐value
rs2195239							
Co‐dominant	CC	183 (50.8%)	179 (42.9%)	1.00		1.00	—
Heterozygote	CG	147 (40.8%)	188 (45.1%)	0.77 (0.57‐1.04)	.085	0.76 (0.57‐1.03)	.077
Homozygote	GG	30 (8.3%)	50 (12.0%)	**0.59 (0.36‐0.97)**	**.036**	**0.58 (0.35‐0.95)**	**.030**
Dominant	CC	183 (50.8%)	179 (42.9%)	1.00	—	1.00	—
	GG + CG	177 (49.2%)	238 (57.1%)	**0.73 (0.55‐0.97)**	**.030**	**0.72 (0.54‐0.96)**	**.026**
Recessive	CG + CC	330 (91.7%)	367 (88.0%)	1.00	—	1.00	—
	GG	30 (8.3%)	50 (12.0%)	0.67 (0.41‐1.07)	.094	0.66 (0.41‐1.06)	.083
Allele	C	513 (71.3%)	546 (65.5%)	1.00	—	—	—
	G	207 (28.8%)	288 (34.5%)	**0.77 (0.62‐0.95)**	**.015**	—	—

Note

The genotyping was successful in 360 cases and 417 controls for rs2195239.

Bold values are regarded as statistically significant.

^a^ Adjustment with age and sex.

Stratified analyses were conducted by sex, age, smoking, and alcohol. IGF‐1 gene rs2195239 polymorphism was related to decreased risk for GC patients among then on‐smokers, non‐drinkers, seronegative *H pylori,* and age <60 years groups (Table 3), indicating that individuals exposing to those factors were not prone to GC.

**Table 3:** Stratified analyses between rs2195239 polymorphisms and the risk of gastric cancer

Variable	rs2195239 (case/control)			CG vs CC	GG vs CC	GG vs CC + CG	GG + CG vs CC
	CC	CG	GG				
Age (y)							
<60	96/96	77/106	11/28	0.73 (0.48‐1.09); .124	**0.39 (0.19‐0.83); .015**	**0.46 (0.22‐0.95); .036**	**0.66 (0.44‐0.97); .004**
≥60	87/83	70/82	19/22	0.83 (0.53‐1.28); .389	0.82 (0.42‐1.63); .579	0.90 (0.47‐1.73); .752	0.82 (0.55‐1.25); .360
Sex							
Male	100/104	79/97	13/97	0.86 (0.57‐1.28); .451	0.59 (0.28‐1.22); .156	0.63 (0.31‐1.28); .204	0.80 (0.55‐1.18); .269
Female	83/75	68/91	17/91	0.68 (0.43‐1.05); .082	0.57 (0.29‐1.13); .105	0.69 (0.36‐1.32); .264	**0.65 (0.43‐0.99); .044**
Smoking							
Yes	114/114	113/83	22/21	1.36 (0.93‐2.00); .116	1.05 (0.55‐2.00); .889	0.91 (0.49‐1.70); .766	1.30 (0.90‐1.87); .161
No	69/65	34/105	6/29	**0.31 (0.18‐0.52); <.001**	**0.26 (0.11‐0.61); .002**	0.45 (0.20‐1.03); .058	**0.30 (0.18‐0.48); <.001**
Alcohol							
Yes	118/86	115/99	22/24	0.85 (0.58‐1.25); .399	0.67 (0.35‐1.27); .218	0.73 (0.40‐1.34); .307	0.81 (0.56‐1.18); .269
No	65/93	32/89	8/26	**0.52 (0.31‐0.87); .013**	0.44 (0.19‐1.03); .060	0.57 (0.25‐1.32); .190	**0.50 (0.31‐0.81); .005**
*H pylori*							
Seropositive	113/86	110/93	22/22	0.91 (0.41‐1.35); .639	0.76 (0.40‐1.46); .413	0.80 (0.43‐1.49); .478	0.88 (0.61‐1.28); .510
Seronegative	70/93	37/95	8/28	**0.52 (0.32‐0.85); .009**	**0.38 (0.16‐0.88); .025**	0.50 (0.22‐1.14); .100	**0.49 (0.31‐0.77); .002**

Note

Bold values are statistically significant (*P* < .05).

### 3.3 Association between IGF‐1 gene rs2195239 polymorphism and clinical features

Next, we explored the relationship between IGF‐1 gene rs2195239 polymorphism and clinical features of GC patients (Table 4). The IGF‐1 gene rs2195239 polymorphism was associated with the tumor size, TNM stage, and adenocarcinoma among GC patients.

**Table 4:** The associations between IGF‐1 rs2195239 polymorphism and clinical characteristics of gastric cancer

Characteristics	Genotype distributions			
rs2195239	CC	CG	GG	CG + GG
Tumor size				
>4 cm/≤4 cm	106/77	66/81	11/19	77/100
OR (95% CI); *P*‐value	1.0 (reference)	**0.59 (0.38‐0.92); .019**	**0.42 (0.19‐0.94); .030**	**0.56 (0.37‐0.85); .006**
Metastasis				
M1/M0	10/173	11/136	3/27	14/163
OR (95% CI); *P*‐value	1.0 (reference)	0.40 (0.58‐3.39); .455	1.92 (0.50‐7.43); .336	1.49 (0.64‐3.44); .352
Histological grade				
PD/WD	61/35	48/17	6/5	54/22
OR (95% CI); *P*‐value	1.0 (reference)	1.62 (0.81‐3.24); .170	0.69 (0.20‐2.42); .559	1.41 (0.74‐2.69); .299
Histological grade				
PD/MD	61/87	48/82	6/19	54/101
OR (95% CI); *P*‐value	1.0 (reference)	0.84 (0.52‐1.35); .464	0.45 (0.17‐1.19); .102	0.76 (0.48‐1.21); .253
R classification				
R2/R1	32/98	17/89	7/16	24/105
OR (95% CI); *P*‐value	1.0 (reference)	0.59 (0.30‐1.13); .106	1.34 (0.51‐3.55); .555	0.70 (0.39‐1.27); .240
R classification				
R2/R0	32/53	17/41	7/7	24/48
OR (95% CI); *P*‐value	1.0 (reference)	0.69 (0.35‐1.41); .302	1.66 (0.53‐5.16); .381	0.83 (0.43‐1.60); .574
Location				
Cardia/Non‐cardia	47/136	50/97	8/22	58/119
OR (95% CI); *P*‐value	1.0 (reference)	1.49 (0.93‐2.40); .099	1.05 (0.44‐2.52); .909	1.41 (0.89‐2.23); .139
TNM				
Ⅲ+Ⅳ/Ⅰ+Ⅱ	90/93	55/92	8/22	63/114
OR (95% CI); *P*‐value	1.0 (reference)	**0.62 (0.40‐0.96); .032**	**0.38 (0.16‐0.89); .022**	**0.57 (0.37‐0.87); .009**
Histology				
Adenocarcinoma/NOT	140/7	142/5	25/5	167/10
OR (95% CI); *P*‐value	1.0 (reference)	1.42 (0.44‐4.58); .556	**0.25 (0.07‐0.85); .018**	0.84 (0.31‐2.25); .721

Note

Bold values are statistically significant (*P* < .05). R0: no cancer infiltration at the margin; R1: microscopic cancer infiltration; R2: macroscopic cancer infiltration.

Abbreviations: MD, moderately differentiation; PD, poorly differentiation; WD, well differentiation.

## 4 DISCUSSION

In this study, data indicated that IGF‐1 gene rs2195239 polymorphism was related to decreased risk for GC in Chinese individuals. In addition, IGF‐1 gene rs2195239 polymorphism conferred decreased risk for GC patients among the non‐smokers, non‐drinkers, seronegative *H pylori*, and age <60 years groups. Furthermore, rs2195239 polymorphism was related to the tumor size, TNM stage, and adenocarcinoma among GC patients.

Recently, a host of studies explored the potential association between IGF‐1 gene rs2195239 polymorphism and various cancer risks. However, they obtained conflicting findings. Canzian et al^20^ firstly investigated the relationship between rs2195239 polymorphism and breast cancer (BC) risk in a Caucasian population. They showed that rs2195239 polymorphism was not related to BC risk.^20^ Another study from America^13^ replicated above negative results. Although no associations were found between BC risk and IGF‐1 gene rs2195239 polymorphism in a Chinese study, they indicated that this SNP influenced on IGF‐I activity in local tissues of BC.^21^ They also suggested that another SNP of IGF‐I gene (rs7965399) was associated with the risk of BC.^21^ Two studies from Japan and America investigated IGF‐1 gene rs2195239 polymorphism in pancreatic cancer, but with inconsistent findings. Nakao et al^22^ showed that this SNP was not related to pancreatic cancer susceptibility, while Dong et al^13^ found rs2195239 polymorphism decreased the risk of pancreatic cancer. The gene functional polymorphism distribution among different races, various sample sizes, genetic heterogeneity, and clinical heterogeneity may contribute to these disaccords. No significant associations were also indicated in other cancers including prostate cancer,^23^ testicular germ cell tumors,^24^ and multiple myeloma.^11^ Regarding GC, only one study from Japan^14^ has addressed the relationship between IGF‐1 gene rs2195239 polymorphism and GC risk.^25^ However, no association was observed in the Japanese population.^25^ In this study, we showed that IGF‐1 gene rs2195239 polymorphism was related to decreased risk for GC in Chinese Han population, which was in accord with the findings of a recent meta‐analysis by Xu et al.^26^ Xu et al found that rs2195239 polymorphism reduced the overall cancer risk, as well as decreasing cancer risk in Asian populations.^26^ It is obvious that the findings of this study were different from those of the Japanese study.^25^ Potential factor may contribute to their conflicting results. First, genetic heterogeneity for GC existed in Chinese and Japanese races. Third, clinical heterogeneity was unnegligible factors. Fourth, the sample sizes varied among this study and the Japanese study. Additionally, subgroup analysis found that IGF‐1 gene rs2195239 polymorphism was related to decreased risk for GC patients among the non‐smokers, non‐drinkers, seronegative *H pylori*, and age <60 years groups. Furthermore, the genotypes of IGF‐1 gene rs2195239 polymorphism were related to the tumor size, tumor clinical stage, and pathological types for GC patients.

Several limitations of this study need to be addressed. First, the sample size was not large enough. Second, the functional significance of other SNPs was unknown and the study does not include any other SNPs. Third, information about expose factors was not enough. Fourth, whether the interaction between *H pylori* infection and IGF‐1 gene rs2195239 polymorphism accounted for decreased risk of GC should be studied. Last, this study is lack of a replication sample set to confirm the positive findings.

In conclusion, IGF‐1 gene rs2195239 polymorphism is associated with decreased risk for GC in this Chinese population. Further studies in other populations with larger sample sizes are warranted.

## AUTHOR CONTRIBUTIONS

Pengli Wang and Xiaoyan Zhou conceived of the study and participated in its design. Pengli Wang and Xiaoyan Zhou conducted the systematic literature review. Xiaoyan Zhou performed data analyses. Pengli Wang and Xiaoyan Zhou drafted the manuscript. All gave final approval and agree to be accountable for all aspects of work ensuring integrity and accuracy.

## STATEMENT OF HUMAN AND ANIMAL RIGHTS

All procedures were in line with the ethical standards of our hospital and with the 1964 Helsinki Declaration.

## INFORMED CONSENT

Informed consents were obtained from the study participants.
